# State of the Art in Tumor Antigen and Biomarker Discovery

**DOI:** 10.3390/cancers3022554

**Published:** 2011-06-09

**Authors:** Klervi Even-Desrumeaux, Daniel Baty, Patrick Chames

**Affiliations:** INSERM U624, 163 avenue de Luminy, 13288 Marseille Cedex 09, France; E-Mails: klervi.even@inserm.fr (K.E.-D.); daniel.baty@inserm.fr (D.B.)

**Keywords:** tumor antigen, biomarkers, immunotherapy, cancer, proteomics

## Abstract

Our knowledge of tumor immunology has resulted in multiple approaches for the treatment of cancer. However, a gap between research of new tumors markers and development of immunotherapy has been established and very few markers exist that can be used for treatment. The challenge is now to discover new targets for active and passive immunotherapy. This review aims at describing recent advances in biomarkers and tumor antigen discovery in terms of antigen nature and localization, and is highlighting the most recent approaches used for their discovery including “omics” technology.

## 1. Introduction

Cancer remains the major devastating disease throughout the world. Cancers are responsible for over 6 million deaths per year worldwide with at least 10 million new cases annually. In developing countries, cancer is the second most common cause of death, comprising 23%–25% of total mortality. Current treatments include chemotherapy and radiotherapy but these are often characterized by a low efficiency and a high level of toxicity.

More targeted therapies are eagerly awaited. Among them, immunotherapies, including any approach aiming at triggering an immune response toward tumor cells, are being actively pursued. The immune system is trained at recognizing and destroying non-self, such as pathogens and transformed cells. However, the immune system is much more efficient at recognizing and attacking germs than cancer cells. In many cases, differences between normal and cancer cells remain subtle and the immune system may not always recognize cancer cells as foreign. Moreover, cancer cells have evolved several strategies to dampen or evade immune responses, leading to cancer spread in the presence of a healthy, working immune system. To overcome this issue, researchers are studying several ways to help the immune system to recognize and destroy cancer cells. Two main types of immunotherapy can be distinguished [1]. Active immunotherapies aim at stimulating the patient's immune system to fight the disease. Passive immunotherapies do not rely on patient's bodies to initiate the immune response but rely on the use of man-made immune components, such as antibodies.

Monoclonal antibodies (mAbs) are the most common form of passive cancer immunotherapy [2]. Once antibodies are injected, they are retained at the tumor site because of their affinity for a tumor antigen. Their mode of action can be direct (for example via induction of apoptosis) or indirect, via the recruitment of effector cells or through the activation of the complement cascade leading to tumor cell lysis. These latter two modes of action are referred to as antibody-dependent cell-mediated cytotoxicity (ADCC) and complement dependent cytotoxicity (CDC) and are mediated through the Fc portion of mAbs. In the case of soluble antigens, mAbs can sequester the target and avoid their interaction with their receptor. From 1980 to 2010, several hundreds of therapeutic mAb have been studied in clinical trials by commercial companies worldwide for a variety of cancer indications. To date, 11 anticancer mAb have been approved by the US FDA for marketing.

Active immunotherapies are mainly developed as cancer vaccines [3]. Unlike regular vaccines, most cancer vaccines are not designed to prevent diseases but rather aim at raising a specific immune response against existing tumor cells. Cancer vaccines may contain cancer cells, parts of cells, or pure antigens. Interestingly, because a successful immune response is generating memory cells capable of being rapidly activated to destroy the same kind of cells, cancer vaccines have the potential to prevent relapses. Cancer vaccines have been studied for several decades, but advances in this field have been slower than for other forms of immunotherapy. However, several experimental treatments are currently leading to encouraging results. And recently, a prostate cancer vaccine has been approved by the FDA to treat advanced prostate cancer [4,5].

The treatment of cancer remains a formidable challenge owing to factors such as difficulties in differentiating tumor cells from healthy cells to fight the disease without causing intolerable toxicity. Much has changed in the last years due to the significant progress in immunology, molecular biology and completion of human genome sequence. Substantial antigenic differences have been found between tumors and normal tissues. A milestone in tumor immunology was the cloning of tumor antigen MAGE-1 by Boon's team in 1991 [6], and subsequent characterization of the first HLA-restricted T cell defined antigenic epitope a year later [7]. Because tumor-specific antigens are exclusively expressed by cancer cells and are often critical for tumorigenicity, they are ideal targets for anti-cancer therapy. However, targeting tumor-specific antigens would require therapeutic strategies to be made to individual patients or small subgroups of patients. Thus, until now mainly tumor-associated shared antigens have been targeted by active and passive cancer immunotherapy. Identification of new tumor antigens may lead to the development of future antigen-specific immunotherapy to tumors. Interestingly, such tumor antigens are often released in the circulation and can be used as biomarkers. More generally, cancer biomarkers can be defined as markers produced either by the tumor itself or by other tissues, in response to the presence of cancer or other associated conditions, such as inflammation. Biomarkers might be DNA, mRNA, proteins, metabolites, or processes such as apoptosis, angiogenesis or proliferation. Such biomarkers can be found in a variety of fluids, tissues and cell lines. They are commonly employed in clinical diagnosis. For example, they can be used to diagnose cancer in an early phase, to subtype within a disease category and to monitor patients for response to therapy. Over the past several decades, enormous efforts have been made to screen and characterize useful cancer biomarkers. Some important molecules including carcinoembryonic antigen (CEA), human epidermal growth factor receptor-2 (HER2/neu), prostate specific antigen (PSA), alpha-fetoprotein (AFP), cancer antigen or carbohydrate antigen (CA 125, CA 15-3 and CA 19-9), have been identified. Interestingly, several biomarkers including HER2, CEA, PSA, mucin-1 (MUC-1) are also used in immunotherapy as tumor antigens.

Immunotherapy has been studied for several decades and has led to several encouraging result. The discovery of new tumor antigens could help to expand these approaches to a wider variety of cancers. Moreover, it is also important to discover new markers for other clinical applications such as diagnosis and prognosis. This review aims at describing the state of the art on current tumor antigens used in immunotherapy, and highlights the recent advances in biomarker and tumor antigen discovery approaches.

## 2. Antigens Used in Immunotherapy

### 2.1. Tumor Antigens: Definition

Based on qualitative differences, tumor antigens are divided into two classes: Tumor-specific antigens (TSAs) that are caused by mutations and tumor-associated antigens (TAAs) that result from over- or aberrant expression of non-mutated proteins.

TAAs represent a group of normal non-mutant molecules that can be subdivided into four major categories according to expression pattern [8]: (1) Cancer-testis like antigens (CT antigens): CT antigens include MAGE-1 [6,9], MAGE-2, MAGE-3, MAGE-12, BAGE, GAGE, NY-ESO-1, and CML66, and CML28 [10]. Cancer-testis antigen are expressed in a wide range of different cancers, but are generally not expressed in most other normal somatic tissues, except testis [11]. Since testis is an immune privileged site that does not express MHC class I or II molecules [12], these antigens can practically be regarded as tumor-specific and are highly desirable as targets for antigen-specific immunotherapy; (2) Differentiation antigens: Differentiation antigens are tyrosinase, TRP-1, TRP-2, gp100, MART-1, CD20, epithelial cell adhesion molecule (EpCAM) and MC1R [13,14]. Since these differentiation antigens are expressed in differentiation stage-dependent and tissue-specific manners, immunotherapy based on these antigens may not cause any side-effects on the other tissues; (3) Oncofoetal antigens: These antigens are found on embryonic and fetal tissues as well as certain cancers. This category includes CEA, α-fetoprotein, 5T4, onco-trophoblast, and solid tumor associated glycoprotein [8,15]; (4) Overexpressed antigens: These antigens are normal proteins whose expression is up-regulated in cancer cells. Examples include PSA, prostatic acid phosphatase (PAP), proteinase 3 (myeloblastin), WT-1, MUC-1, wild-type p53, Her2/Neu, G250, prostate specific membrane antigen (PSMA) and epidermal growth factor receptor (EGFR) [16-19]. Because of their expression in normal tissue, TAAs are more likely to have induced immunologic tolerance [20]. Self-reactive T cells are deleted or inactivated, and when not deleted, have a reduced capacity to recognize target antigens [21]. If a cancer vaccine does break tolerance to TAAs, destruction of normal tissues or even fatal autoimmune damage might result from the generation of self-reactive T cells [22,23].

The second group of antigens is TSAs. Cancer results from the accumulation of somatic mutations, and cancer cells contain a large number of mutant proteins [24] than can be recognized as TSAs in an individual patient. With the large number of mutations found in common human cancers [25,26], every human cancer cell should harbor at least few mutations that can be therapeutically exploited, if the corresponding peptide can be efficiently presented by HLA molecules. TSAs are ideal targets for cancer immunotherapy because they are exclusively expressed by cancer cells and not on non-malignant tissues, minimizing the risk of autoimmune destruction. During tumor development, the immune system can recognize these determinants as non-self and generate specific high-affinity antibodies and T cells against them. Advantages of tumor-specific antigens include immunogenicity, decreased risk of inducing autoimmunity, decreased risk of immune escape and immunodominance, which make tumor-specific antigens attractive targets for immunotherapy. However, unlike TAAs, TSAs are expressed only on individual patient's cancer cells or small subsets of tumors and thus require the development of personalized therapy. Examples of TSA include three Ki-RAS point mutations (single amino acid substitutions) that are found in about 95% of all patients with pancreatic cancer. Another example can be given with mutated p53. The pivotal role of p53 as a tumor suppressor is illustrated by the fact that this protein is found mutated in ∼50% of human cancers. In most cases, mutations in p53 greatly increase the otherwise short half life of this protein and cause it to accumulate in tumor cells. The aberrant p53 expression in many malignancies offers an attractive opportunity for antigen-specific immunotherapy of cancer [27]. This is because the mutated p53 that is present in tumor cells may be considered “nonself” or tumor specific [28]. The tumor specific mutations present in the p53 protein may alter its antigenicity, if the mutations occur in a region of the protein that can be presented as an epitope to the T cell.

Over the last 10 years, the genetic origins of several TSAs have been identified but in each case the mutations identified were only found in one individual tumor but not in cancer cells from other patients [29,30]. However, once more cancers are analyzed, prominent target genes and mutation patterns will likely emerge. Improvements in “omics” technology and database information should soon make such individually tailored therapies a reality.

### 2.2. Passive Immunotherapy

Passive immunotherapies use immune system components such as mAbs to attack the disease [31]. Naked mAbs are currently the most commonly used mAbs. Although they all work by binding to specific antigens, they use various mode of action. Some naked mAbs bind cancer cells to act as a marker for the body's immune system to destroy them. Such approved antibodies include: Rituximab, Ofatumumab and Alemtuzumab (Table 1). The effects of other naked antibodies come from their ability to bind to some receptors or ligands, thereby blocking their interactions with their cognate ligand or receptor, and avoiding some signaling necessary to the proliferation of cancer cells. Examples of FDA-approved mAbs of this type include: Trastuzumab, Cetuximab, Panitumumab and Bevacizumab (Table 1). By contrast, conjugated mAbs are monoclonal antibodies that are linked to drugs, toxins, or radioactive substances. The mAbs are used as transporters to deliver these substances directly to cancer cells. Conjugated mAbs can be divided into groups depending on what they are linked to. MAbs linked to radioactive particles are referred to as radiolabeled, and therapy with this type of antibody is known as radioimmunotherapy (RIT). Two radiolabeled antibodies have been approved to treat cancer: Ibritumomab tiuxetan and Tositumomab (Table 1). MAbs linked to chemotherapy drugs attached are often referred to as chemolabeled and mAbs linked to toxins are called immunotoxins. There are no chemolabeled or immunotoxins approved for cancer therapy so far.

The efficacy of anti-cancer mAbs is critically dependent on the nature of the target. An ideal tumor cell surface target should be accessible, abundant, homogeneous and consistently present on the surface of cancer cells within a tumor [32]. Importantly, targets should not be expressed on normal cells, especially those that constitute vital organs, so that anti-cancer mAbs can discriminate between healthy and malignant cells. Ideally, targets should not be secreted in any form by the tumor cells into the circulation because anti-cancer mAbs might bind to the soluble circulating antigen rather than the antigen presented by tumor cells. If ADCC or CDC modes of action are desired, the antigen-mAb complex should not be rapidly internalized by the cell because the Fc portion cannot activate the immune system. By contrast, internalization is necessary for cytotoxic activity in the case of some immunotoxins. These targets must be capable of antibody mediated internalization, or have an intrinsically high turnover rate. Candidate therapeutic mAbs currently investigated in clinical studies are targeting approximately 80 different antigens (Table 2) [33]. However, only 10 different antigens are currently being targeted by mAbs developed for cancer therapies: EpCAM, MUC1, EGFR, CD20, CEA, HER2, CD22, CD33, Lewis Y and PSMA. The small size of this set clearly highlights the necessity to discover new tumor targets.

### 2.3. Active Immunotherapy

During the last decades, various strategies have been proposed to overcome the poor immune response against TAAs, including cell-based vaccines, DNA- or RNA-based vaccines, protein or peptides based vaccines, and vector based vaccines [34]. The common rational for all these modalities is the activation of antigen presenting cells (APCs) and the stimulation of an antigen-specific cytotoxic T lymphocyte (CTL) mediated immune response (Table 3).

A first vaccination strategy relies on the use of vectors. Several vectors can be used to deliver recombinant genes (including genes expressing TAAs, costimulatory molecules, or cytokines) into APCs. Recombinant vector-based vaccines may induce the immune system to generate a response against the genes of interest that have been inserted into the vector. One advantage of using vectors as vehicles for TAAs is that this type of delivery of a recombinant protein is much more immunogenic than the administration of the protein with adjuvants [35]. Vectors used in cancer immunotherapy include viral, bacterial, and yeast vectors. Poxviral vectors are among the most heavily exploited in vaccine development. The large genome of poxviruses (approximately 130 kb for mammalian poxviruses and 300 kb for avian poxviruses) allows for insertion of more than 10 kb of foreign DNA. Moreover, gene products are usually expressed at high levels, resulting in a potent cellular immune response. Two vector vaccines are actually in clinical trials: PSA-TRICOM vaccine (prostate-specific antigen plus a TRIad of Costimulatory Molecules; PROSTVAC) [36-39] and PANVAC-VF, another poxviral-based vaccine that consists of a priming vaccination with recombinant vaccinia encoding CEA(6D), MUC1(L93), and TRICOM plus booster vaccinations with recombinant fowlpox expressing the identical transgenes [40-42]. TG4010 is another vaccine. It incorporates the MUC1 antigen, which is overexpressed in the majority of cancers, into a non-propagative pox viral vector, MVA. A second gene, interleukin-2 is also incorporated into TG4010 as an immune stimulus. The vaccine has been tested in breast, kidney, prostate and lung cancers with encouraging results [43-45] (Table 3).

The second method relies on the use of proteins or peptides to stimulate a specific immune response against cancer and employs single agents or combinations of proteins, heat-shock proteins (HSPs), peptides and agonist peptides, antiidiotype antibodies, and fusion proteins. These protein- or epitope-based vaccines have two main advantages over the use of tumor cells or lysates: Production, storage, and distribution are faster and more cost-effective, and the identification and administration of TSAs is preferable since tumor-cell preparations mostly contain self-proteins with no therapeutic benefit and are potentially capable of generating an autoimmune response. On the other hand, this approach has certain drawbacks: Single protein or, especially, a single epitope are sometimes weakly immunogenic. Tumors can easily escape immune recognition through antigen mutation. Their use is HLA restricted (mainly for epitope-based vaccines) and limited to a subset of patients (usually HLA-A2+). They have a poor ability to induce balanced activation of CD4 and CD8 subsets, which is thought to be essential for effective antitumor immunity. The use of specific proteins or peptides as targets for immunotherapy clearly requires a careful choice of the targeted TSAs or TAAs and their epitopes, involving knowledge of their structural and functional characteristics. Single-peptide epitope composed of 8 to 10 amino acids are able to induce a CTL response by binding to MHC class I molecules expressed on APCs. Several antigen vaccines are actually in clinical trials (Table 3) [4,5,46-48]. Provenge (sipuleucel-T, Dendreon Corporation), recently approved by FDA, is an autologous cellular immunotherapy from T cells designed to stimulate T-cell immunity against prostatic acid phosphatase (PAP) [4,5] (Table 3). Stimuvax (BLP25 liposome vaccine, L-BLP25, Oncothyreon partnered with Merck KGaA) is a cancer vaccine designed to induce an immune response against the extracellular core peptide of MUC1, a type I membrane glycoprotein widely expressed on many tumors (*i.e.*, lung cancer, breast cancer, prostate cancer, and colorectal cancer) [46] (Table 3). To avoid the disadvantages of using short peptides, the concept of synthetic long peptides (SLP) has been developed as vaccines [49]. When injected, these SLPs are predominantly taken up by dendritic cells resulting in the presentation of both helper T-cell epitopes and CTL epitopes that are present in the SLP [50]. In a recent study, a p53-SLP vaccine was found capable of inducing p53-specific T-cell responses in patients treated for colorectal cancer [27].

The third strategy of vaccine is the use of tumor-cells or lysates [40,51,52]. Tumor-cell vaccines have at least three advantages over the single-target approaches in terms of eliciting an immune response: Different and unknown antigens can be targeted at the same time, the immune response is not HLA-restricted, the variety of both MHC class I and class II epitopes processed is likely to be able to stimulate both an innate (natural killer cells, macrophages, and eosinophils) and adaptive (CD8+ and CD4+ T cells) response. The first important distinction is between vaccines using autologous (patient-specific) or allogeneic (non patient-specific) tumor cells. Second, these cells may be unmodified, modified for expression of MHC, costimulatory molecules, or cytokines, or used in combination with adjuvants such as GM-CSF and Bacille Calmette-Guerin (BCG). Third, these cells can be used in the form of tumor-cell lysates [52]. In the past 20 years, several different vaccines derived from whole tumor cells or tumor-cell lysates have been evaluated in preclinical models and clinical trials. OncoVAX (Vaccinogen) is composed of autologous irradiated tumor cells, with or without BCG as an adjuvant [52]. Reniale (LipoNova) is a vaccine based on a lysate of autologous tumor cells, preincubated with IFN-*γ* to increase the antigenicity of these cells, and tocopherol acetate to protect cell membranes during the incubation process [51,52] (Table 3).

The last strategy is DNA- or RNA-vaccines. In this case, cells are injected with DNA encoding protein antigens. DNA-based vaccines are a recently developed strategy that has proven capable of activating strong immunity against weak TAAs. Recently, several phase I/II clinical trials employing DNA-based vaccines targeting different TAAs (*i.e.*, PSA, PAP, gp100, CEA, hsp65) have been conducted in patients with prostate cancer, melanoma, colorectal cancer, and head and neck carcinomas [53-57]. The mRNA-based vaccine containing the mRNA-coding TAA is transfected into DCs and translated into proteins. After protein processing, the antigen can be loaded on MHC molecules for antigen presentation, thus activating an antigen-specific CTL response. Clinical trials have been performed employing mRNA transfected DCs or injecting mRNA directly into patients with prostate cancer, renal cancer, ovarian cancer, lung cancer, breast cancer, pediatric brain cancer, neuroblastome, and melanoma [58-63]. A phase I clinical trial was performed using PSA-mRNA transfected DCs in patients with metastatic prostate cancer [64] (Table 3).

### 2.4. Glycoproteins: A New Avenue

Until recently, tumor-specific tumor antigens that have been identified in mouse and human are mutant peptide epitopes. In eukaryotic cells, 95% of all proteins are post-translationally modified and glycosylation is the most frequent post-translational modification found. It is estimated that 50%–80% of cellular proteins—Membrane, cytosolic and nuclear—are glycosylated [65]. Although the amino acid sequence of proteins predominantly determines their three-dimensional structure, the post-translational modifications of the proteins modulate their physical and chemical properties and thus their stability and molecular function. Since glycoproteins, carbohydrates and glycolipids are the most abundant structures present on the surface of eukaryotic and prokaryotic cells, they are the first structures encountered by the immune system. Initially, it was thought that only pure protein epitopes could be presented on MHC and induces T cell responses. This was due in part to the observation that immunization with carbohydrate antigens usually resulted in low-affinity IgM responses without memory. To obtain a strong immune response, as reflected by IgG production, both T and B cells are required. However, recent publications have demonstrated that non-peptide molecules such as pure carbohydrates, glycopeptides and glycolipids can be presented on MHC molecules and recognized by T cells [66-73]. Although pure peptide epitopes are still considered as the primary targets for T cell responses, there is agreement that glycopeptides also induce T cell responses [68,70,72,73]. Therefore, the peptide epitopes that have been identified thus far as tumor epitopes might represent only a small fraction of potential targets. There are two types of glycosylation, *N*- and *O*-glycosylation [74,75]. *N*-Glycosylation occurs at the amino acid asparagine (Asn). The consensus sequence for *N*-glycosylation is the presence of the amino acid sequence Asn-*X*-serine/threonine (*X* may be any amino acid except proline). *O*-Glycosylation occurs at serine (Ser) or threonine (Thr) residues, but despite much effort, no consensus sequence for *O*-glycosylation has been identified. Aberrant glycosylation has been recognized for more than 30 years as a typical feature of cancer [74-80]. Changes in cell surface carbohydrate structures occur during tumor progression, invasion and metastasis [81]. Cancer cells frequently display glycoproteins with increased branching of the glycan structures and/or altered expression levels compared with normal cells [82]. Such aberrations occur in both *N*- and *O*-linked glycosylation. Due to their wide expression profile in several malignancies, much effort has gone into targeting tumor-associated carbohydrate antigens (TACAs) with active and passive immunotherapy and trying to augment their antigenicity and immunogenicity [83-85]. In addition to TACAs, gangliosides (GD3, GD2 and GM2) have also emerged as promising mAb targets for various cancers such as melanoma and neuroblastoma [78,86]. Intriguingly, many of the oldest and most widely used clinical cancer biomarker tests detect glycoproteins. These include CEA, commonly used as a marker of colorectal cancer, CA 125, frequently used to diagnose ovarian cancer and PSA for prostate cancer [87-93].

### 2.5. Intact Intracellular Proteins as Tumor Antigens: The Intrabody Concept

An intrabody (for intracellular antibody) is an antibody that has been designed to be expressed intracellularly, opening the possibility to specifically block a precise interaction of a antigen into the intracellular compartments of living cells. Intrabodies can be directed to a specific target antigen present in various subcellular locations including the cytosol, nucleus, endoplasmic reticulum (ER), mitochondria and trans-Golgi network (TGN). Combining specificity and antigen-binding affinity, intrabodies have been used as a biotechnological tool to interrupt, modulate, or define the functions of a wide range of target antigens at the posttranslational level. These intracellular antibodies are being developed to bind to, neutralize, or modify the function or localization of cancer-related targets and thereby affect the malignant phenotype.

There are many ways in which intrabodies could be used inside the cell to affect protein function [94,95]. Apart from blocking protein-protein or protein-nucleic acid interactions [96], it is possible to design intrabodies that bind antigen and relocate it to an inappropriate subcellular location. Intrabodies can also be employed to inhibit directly the function of an enzyme [97-99], or even to promote the death of target cells (e.g., cancer cells) by inducing caspase-3-mediated apoptosis [100]. In the field of cancer, intrabodies have been used to modulate the expression of proteins upregulated in tumors, such as erbB-2, interleukin-2 receptor, cyclin E (cell cycle protein), and EGFR [100-109]. In all these cases, appropriate cellular localization signals were fused to the intrabodies to reduce the activity of tumor-related proteins by altering their location. Oncogenic proteins, such as tumor protein 53 (p53) and proto-oncogen (RAS) [110], which are mutated in a large number of tumors are good candidates for intrabody therapy because they are tumor-specific therapeutic targets. A major challenge for the successful application of intrabodies for therapy is achieving sufficient internalization or expression inside target cells. Introducing intrabodies *in vitro* into cell lines in tissue can be achieved via gene expression using standard methods or by use of protein transduction domains linked to intrabodies [111]. Intrabody delivery *in vivo* represents another level of difficulty. Virally mediated gene transfer is a good option or an alternative approach would be to use immunoliposomes [112-114].

### 2.6. Biomarkers

A biomarker, according to the US national Cancer Institute, is a biological molecule found in blood, another body fluid or in tissues that is a sign of a normal or abnormal process. Generally, biomarkers are produced by either the tumor itself or other tissues, in response to the presence of cancer or other associated conditions. Historically, cancer protein biomarkers have been discovered in body fluids and tumor tissues (or cell lines) using two dimensional polyacrylamide gel electrophoresis (2D-PAGE) separations or by identifying immunogenic antigens on cancer cells [115]. Conventional approaches have successfully produced FDA-approved blood-based cancer biomarkers and most of which are used to monitor treatment [116] (Table 4). Tumor markers can be used for screening of a general population, for differential diagnosis in symptomatic patients, and for clinical staging of cancer. A number of different types and forms of tumor markers exist. These markers include hormones, as well as different functional subgroups of proteins such as enzymes, glycoproteins, oncofetal antigens and receptors.

Importantly, a number of biomarkers used in diagnosis are also tumor antigens used in cancer therapies. Relevant examples include CEA, HER2 and MUC-1 [117-19] (Table 4). These molecules are membrane proteins targeted use in immunotherapy. However, the extracellular domain of these proteins is shed into the bloodstream and can be detected in serum. Consequently, the discovery of new biomarkers for diagnostic purposes might also in some case be of high interest for the discovery of new tumor target for therapeutic approaches.

## 3. Strategies of Discovery of New T-cell Antigen and Biomarkers

Cancer is a complex disease that reflects genetic, as well as protein changes within a cell. During the past two decades, there has been a growing interest in approaches for discovering new biomarkers that may allow identification of potential targets for drug therapy. New biomarkers are urgently needed to accelerate efforts in developing new drugs and treatments of diseases. The explosion of high-throughput technologies available for generating large-scale molecular-level measurements in human populations has led to an increased interest in the discovery and validation of molecular biomarkers in medical research. Most biomarkers and tumoral antigen are not satisfactory because of their limited specificity and/or sensitivity. So, there is an urgent need to discover better targets in clinical practice. Global gene expression analysis has been extensively utilized, and the cancer management results are currently being translated into clinical tests, such as MammaPrint [120] and Oncotype DX [121] used for breast cancer. But gene expression data gives limited information since proteins are the main functional units performing all biological process in the cell or organism and may have post-transcriptional event(s) and post-translational modification(s) that contribute to the biological activity of proteins. The direct analysis of protein, the functional unit of the cell, using proteomics analysis has several advantages despite requiring more tissue and being more time-consuming. Several proteomics technologies including 2D-PAGE [122-124], surface enhanced laser desorption/ionisation time of flight (SELDI-ToF) [125], protein arrays [126,127], isotope coded affinity tags (ICAT), iTRAQ and multidimensional protein identification technology (MudPIT) are the approaches being implemented in cancer research (Table 5). 2D-PAGE and SELDI-ToF are the main technologies used in serum cancer research. However other technologies such as protein arrays, ICAT, iTRAQ and MudPIT also offer great potential for future biomarker discovery in cancer.

### 3.1. T-cell Antigens

One of the major contributions that proteomics has made to the medical and pharmaceutical community is the identification of potential drug targets. The identification and molecular characterization of self antigens expressed by human malignancies that are capable of elicitation of anti-tumor immune responses in patients has been an active field in tumor immunology. Much has changed in the last twenty years due to the significant progress in immunology, molecular biology and completion of human genome sequencing [128-130]. Substantial antigenic differences have been found between tumors and normal tissues. A great deal of evidence in mice and men has demonstrated that the host generates antibodies and T cells against developing tumors. Strategies have been developed to use patient's T cells (CD4+ and CD8+) and IgGs for the identification and characterization of tumor antigens.

Three methods have been developed to define T cell tumor antigens. First method is T cell epitope cloning. cDNA libraries generated from tumor cells are transfected into target cells expressing the appropriate MHC Class I or II molecules, and anti-tumor T cells are used to identify the appropriate transfectant [6,9,131-134]. This method is a labor-intensive process and requires T cell culture and cloning expertise. Second is HLA-binding peptide elution. Peptides are eluted from the surface of cancer cells (or from MHC Class I or II molecules purified from cancer cells), pulsed onto APC and tested for reactivity with the patient's lymphocytes. Purification and sequencing of these peptides identifies the parental protein [135-137]. This method requires protein chemistry expertise in peptide purification and high power mass spectrometry. A third approach consists in identifying a subcellular compartment containing the CD4+ T cell-stimulatory activity, followed by separation of the stimulatory protein fraction by reversed-phase high-performance liquid chromatography (RP-HPLC). The resulted fractions are then subjected by gel electrophoresis. The stimulatory band, identified by T-cell Western blotting, is finally subjected to protein sequencing [138].

A fourth method is to identify TAAs recognized by the antibody repertoire of cancer patients. These TAAs are overexpressed in patients and found at the surface of cells in peptide-MHC complex. TAAs identified with this method could be used for vaccination. In 1995, Pfreundschuh's team developed this method of serological cloning approach called SEREX [129,139-141], which allows a systemic and unbiased search for antibody responses against protein antigens expressed by human tumors. The development of SEREX offered a high-throughput approach to analyze the humoral response against TAAs in cancer patients. This has allowed the direct molecular identification of antigenic tumor proteins. The respective tumor antigens in the recombinant cDNA libraries are identified from their reactivity with antibodies in the autologous and allogeneic sera of cancer patients. The advantages of SEREX include rapid identification of multiple tumor antigens and no need for establishment of tumor cell lines and pre-established CTL clones [129]. SEREX remains the prominent technology for identifying TAAs that could be used for immunotherapy [142] or diagnosis and prognosis [143].

### 3.2. Biomarkers

#### 3.2.1. Sample choice and preparation

Blood is the most commonly used biological fluid for biomarker analysis in clinical practice. The advantages of using blood, serum and plasma as a source to mine for biomarkers include that it can be obtained through a minimally invasive procedure, it is abundantly available and some constituents of blood reflect diverse pathological states. It is known that plasma proteins range in concentration over 12 orders of magnitude and that 99% of the protein mass is comprised of only 22 proteins. For example, the most abundant plasma protein is albumin, which is present in plasma at a concentration of ∼50 mg per milliliter. In contrast, known cancer derived proteins in the circulation are present at a few nanograms per milliliter, 10 million times less abundant than albumin. This large dynamic range of analytes in blood is a major disadvantage for using this source for biomarker discovery. The removal of predominant proteins facilitates better detection of less abundant proteins, but such depletion can lead to the loss of informative molecules. Without fractionation, the complexity of serum and plasma is a limitation, and important biological information can be lost in the background noise [144]. Early experiments in proteomics profiling of serum and plasma made evident that there is no technology platform that can analyze proteins quantitatively with a dynamic range of concentration as high as 10^12^ [145] and that pre-fractionation of these samples is necessary [146,147]. Currently, the major objective of clinical proteomics utilizing body fluids is to reduce the dynamic range of proteins in analyzed samples [145,147]. Initially, columns and cartridges for albumin and IgG were available [148,149] and were soon followed by columns for multiple protein removal, based on immunodepletion [150]. In a relatively short period, removal of most abundant proteins from serum/plasma became a standard first step in clinical proteomics analyses aiming at biomarker discovery [151]. This widely-used approach is now commonly accepted as the first step in sample preparation and it is quite obvious that immunodepletion of the 12 most abundant proteins is necessary (*i.e.*, albumin, IgG, fibrinogen, transferrin, IgA, IgM, haptoglobin, apo A-I, apo A-II, a1-antitrypsin, a1-acid glycoprotein, a2-macroglobulin). These proteins comprise over 96% of total protein content in plasma/serum [145]. However, immunodepletion of multiple proteins can increase the risk of losing proteins of interest or low abundant candidate biomarkers that are removed along with those specifically depleted. Sample preparation remains one of the most time consuming and error prone aspects of analytical chemistry.

Another source of sample is surgically-removed or biopsy-obtained tissues. They are currently being considered as alternative sources for biomarker discovery. One of the major advantages of using tissues is that the concentration of candidate biomarkers should be highest in tumor tissues and they should be a rich source for plasma biomarkers. Under this strategy, candidate biomarkers are first discovered in tumors and then subsequently measured out in the plasma using highly sensitive, targeted assay technologies. Tissues can be difficult to obtain in sufficient quantities, especially normal counterparts for comparative analyses. Alternatively, cancer cell lines can be analyzed. They are easy to handle and comprise a homogeneous and almost inexhaustible source of biological material, including proteins. However, each cell line represents only one tumor unaffected by signals from the microenvironment, and the cells may have been subjected to clonal drift and *in vitro* selection, which may render them less representative of the tumor from which they originated [152-154].

Plasma membrane proteins that are exposed on the cell surface have important biological functions, such as signaling into and out of the cells, ion transport, and cell-cell and cell-matrix interactions. The expression level of many of the plasma membrane proteins involved in these key functions is altered on cancer cells, and these proteins may also be subject to post-translational modification, such as altered phosphorylation and glycosylation. Additional protein alterations on cancer cells confer metastatic capacities, and some of these cell surface proteins have already been successfully targeted by protein drugs, such as mAbs. Because plasma membrane proteins are low-abundant proteins compared with many soluble proteins, the overall fraction of plasma membrane proteins in a cell/tissue lysate is very low, making them difficult to study, even with the recent advances in proteomics technologies [155-157]. The combination of novel analytical approaches and subcellular fractionation procedures has made it possible to study the plasma membrane proteome in more detail, which will elucidate cancer biology, particularly metastasis [158,159], and guide future development of novel drug targets.

There are different strategies of sample preparation based on depletion of highly abundant proteins or on sample fractionation. (1) Centrifugal ultrafiltration is a variation of membrane filtration in which centrifugation forces a liquid against a semi-permeable membrane. Suspended solids and solutes of high molecular weight are retained, while the liquid and low molecular weight solutes pass through the membrane depending on the molecular weight cut off of the membrane used [160,161]; (2) Solid phase extraction (SPE) is a separation technique that uses a solid phase to isolate one, or one type, of analyte from a solution. SPE is commonly used in serum/plasma samples as a clean-up step to remove highly abundant proteins. Solid phase extraction columns are probably the approach most widely used for depletion of high abundance proteins in serum/plasma. Different types of SPE columns based on ion-exchange [162-164], metal chelating, affinity ligands [165], dye-ligands [166-168], bacterial proteins [169,170], antibodies [165,171] or combinations of these have been used. Surface-enhanced laser desorption/ionization (SELDI) is an affinity-based mass spectrometric method that combines sample fractionation with mass spectrometry (MS) analysis. In recent decades, magnetic beads have emerged as a promising new platform in biomedical applications, particularly bioseparations [172]. Functionalized magnetic beads are used for solid phase extraction of a specific subset of molecules from a liquid. The nature of the molecules retained depends directly on the kind of surface-derivatized beads used; (3) The third method is organic solvent extraction. The possibility of selectively removing large abundant proteins from serum by precipitating them with simultaneous extraction of peptides and low molecular weight proteins using organic solvents has been tested [173]. The precipitation with organic solvents in the presence of ion-pairing agents dissociates peptides and smaller proteins from large abundant proteins, thereby facilitating their extraction. Other strategies consist of sample fractionation before analysis by MS such as electrophoresis and chromatographic processes could be considered as sample preparation but also analysis strategies. These methods will be described later in the review. For enrichment of plasma membrane proteins, most strategies used either homogenization followed by membrane density separation or whole cell protein tagging followed by affinity purification. Enrichment of plasma membrane proteins using whole cell protein tagging is often based on a membrane-impermeable biotin labeling reagent followed by cell lysis and affinity purification using streptavidin-coated beads [174-176].

#### 3.2.2. Gene expression profiling

In the late 1990s, DNA microarray technology emerged as a powerful tool for the analysis of the levels of mRNA transcripts expressed under various conditions. For example, microarray technology has been used to compare gene expression profiles in ovarian cancers and normal ovaries. The aim is to identify genes that are differentially expressed between the two states, with the expectation that similar patterns could be seen for the respective proteins in serum [177]. Several studies have attempted to identify new molecular biomarkers for the early detection of ovarian cancer by gene expression profiling [178-180]. The advantages of this approach include high throughput and objective molecular subclassification. Gene expression levels reflect the cumulative effect of several underlying biological functions as DNA-microarray technology has enabled the simultaneous examination of thousands of genes, in contrast to studying the expression of single genes. Current microarray platforms are highly automated and enable parallel sample analysis. Although information on mRNA expression levels and the corresponding protein abundances (or activities) are undoubtedly useful in genomic analyses, their values do not always correlate. Furthermore, the analysis of mRNA transcripts does not provide information regarding post-translational modifications (e.g., proteolysis, phosphorylation, glycosylation, acetylation, and deamination) of target proteins. Thus, alternative analytical methods are necessary for extended proteome studies.

#### 3.2.3. Mass spectrometry

For protein quantification, the most commonly used method has been the ELISA [181] (Table 5). Due to its sensitivity and reliability, this method is widely used both in biomedical research and clinical diagnostics of proteins. Another important method, immunohistochemistry (IHC) is capable of localizing proteins of interest within a cell or tissue utilizing specific visualization techniques, such as fluorescently labeled antibodies [182]. However, this method is not able to quantify the exact amount of the proteins, but multispectral imaging does allow the examination of different proteins in a single measurement. However, these procedures are not applicable for target discovery principally due to the low throughput of the methods and the necessity of large volume of sample (Table 5). For this reason, MS-based protein identification combined with quantitative measurements is at the center of development of new technologies and methods. In MS, proteins are digested to predictable peptide fragments using proteases such as trypsin. Tryptic digests of biological proteomes (e.g., tissue or plasma-derived proteins) can be analyzed using different modes of MS, depending on the desired application. For example, untargeted modes of mass spectrometry are used for *de novo* discovery of biomarker candidates such as from tumor tissues or proximal fluids. In contrast, targeted modes of mass spectrometry allow us to look for peptides (and so proteins) of interest in clinical specimens (SILAC, iTRAQ, ICAT, see later in this review). These modes of mass spectrometry can be very useful for determining whether biomarker candidates discovered in tissues or proximal fluids are present (and elevated) in plasma from cancer patients compared to controls. Moreover, all these techniques appear to be complementary and not exclusive. Mass spectrometers consist of an ionization source, a mass analyzer, and a detector. Although there are a variety of ionization sources (e.g., electrospray and matrix assisted laser desorption ionization) and mass analyzers, all MS instruments have these basic features in common. In a typical analysis of a biological sample, proteins or peptides are introduced into the ionization source where they are converted to gas-phase charged particles (ionized) and passed to the mass analyzer. In the mass analyzer, the ions are separated (using electric and magnetic fields) based on their mass-to-charge (*m/z*) ratios. The detector electrically detects the beam of ions passing through the machine (*i.e.*, the ion current) and amplifies the signal, which is recorded in the form of a mass spectrum. The fragmentation pattern is compared to the theoretical fragmentation pattern for every peptide in the genome to find the closest match. In this way the sequence of the peptide ion is inferred from its fragmentation pattern.

##### 3.2.3.1. Two-dimensional electrophoresis

Electrophoresis is the movement of charged particles through a medium by using an electric field induced by electrodes. In proteomics, electrophoresis, especially gel electrophoresis, is still the most used separation technique for complex protein mixtures. Gel electrophoresis refers to the technique in which molecules are forced across a span of gel motivated by an electrical current. Activated electrodes at either end of the gel provide the driving force. The properties of the molecules, such as size, electric charge, structure, *etc.* determine how rapidly an electric field can move them through the gel. 2D-PAGE is widely used in proteomic studies due to its separation power. Proteins are initially separated according to their isoelectric point (pI) by isoelectric focusin (IEF) in the first dimension, followed by separation in the second dimension according to their molecular weight. The result is an array of spots detected by different staining procedures [183]. Up until now 2D-PAGE has been primarily utilized to analyze complex protein mixtures in most laboratories [122,123]. This method allows comparative studies of different samples, such as normal *versus* diseased, or treated *versus* untreated, in order to determine expressional differences at the individual protein or protein group level, assumed to be responsible for phenotype changes (Table 5). 2D-PAGE is the most widely used proteomics technique to study the proteome as well as cancer biomarkers [184-188]. In a proteomics study of breast cancer serum, two proteins, hsp27 (up-regulated) and 14-3-3 sigma (downregulated) were identified using 2D-PAGE coupled with MALDI-TOF-MS [189]. Another example is identification of potential serum markers in pancreatic cancer. Serum samples from 3 pancreatic cancer patients and 3 normal and healthy individuals were analyzed using two dimensional differential gel electrophoresis (DIGE) coupled with MALDI/TOF/TOF-MS and 24 unique up-regulated proteins and 17 unique downregulated proteins were identified in cancer serum [188]. To identify the proteins of interest, other downstream processes such as Western blotting and/or MS is applied. The resolution of this method is sufficient to separate protein isoforms modified by post-translational processes. However, this approach has several limitations: (a) Difficulty with automation; (b) Poor detection of low-abundance proteins; (c) Difficulty in separating hydrophobic membrane proteins, and basic and high molecular mass proteins; (d) Poor reproducibility; and (e) Time-consuming protocols. Modified 2D electrophoresis by fluorescent tagging of proteins (DIGE), offers increased throughput, ease of use, reproducibility, and accurate quantitation of protein expression differences [190]. This system enables the separation of two or three fluorescently labeled protein samples (Cy2, Cy3 and Cy5) on the same gel.

##### 3.2.3.2. Chromatographic processes

Chromatographic processes can be defined as separation techniques involving mass-transfer between stationary and mobile phases. Liquid chromatography (LC) is the most widely used mode of analytical chromatography and uses a liquid mobile phase to separate the components of a mixture. These components (or analytes) are present in a liquid phase or dissolved in a solvent, and then forced to flow through a chromatographic column usually under high pressure (HPLC). In the column, the mixture is resolved into its components. As a result, LC acquires a high degree of versatility not found in other chromatographic systems and it has the ability to easily separate a wide variety of chemical mixtures. Application of LC–MS to biomarker discovery is not yet very widespread partly because the method generates large and highly complex data sets that require powerful algorithms and software tools to handle and analyze them.

##### 3.2.3.3. SELDI-TOF MS

SELDI-TOF MS, introduced in 1998 by Ciphergen [191] is an innovative microarray approach, and offers on-chip purification of unlabeled target proteins followed by subsequent ionization and MS detection of the retained molecules [192]. This technique allows proteins/peptides to be profiled from different biological samples on a variety of chemically (e.g., anionic, cationic, hydrophobic, hydrophilic, metal affinity capture) or biochemically (e.g., immobilized antibody, receptor, DNA, enzyme) defined chromatographic surfaces (Table 5). A small amount of sample of interest is loaded onto ProteinChip™ arrays that selectively bind different subsets of proteins in crude samples by adsorption, partition, electrostatic interaction or affinity chromatography according to their surface chemistries. After a short incubation period, unbound proteins and unspecific substances are washed away with an appropriate buffer and water. The ToF reader records the time-of-flight and calculates the accurate molecular weight of proteins/peptides in the form of a spectral map containing mass to charge ratios (*m/z*) and intensities corresponding to each bound protein/peptide. For example, applications of SELDI-ToF have been demonstrated for the early detection of prostrate [193,194], breast [195,196] and pancreatic [197] cancer biomarkers. SELDI was also used in the discovery and detection of a number of cancer-associated biomarkers, including those for ovarian cancer [198], prostate cancer [199] and breast cancer [200]. However, there is some controversy over this technology such as its reproducibility, the bioinformatics used, the possibility of over-fitting, the potential bias in the samples, as well as how this could possibly fit into a routine diagnostic lab [201,202].

##### 3.2.3.4. Laser capture microdissection

Analysis of human tissue is essential for translational research because cell cultures and even animal carcinogenesis models may not accurately represent the complexities of human disease states [203]. Laser capture microdissection allows scientists to procure pure cell populations from heterogeneous tissue sections [204,205]. Protein or DNA/RNA may be analyzed from the microdissected cells, lending this technology to comprehensive molecular profiling of tissues.

Laser-capture microdissection, described by Emmert-Buck *et al.* in 1996 brings molecular analysis to the cellular level [204]. This technique allows for precise collection of pure cell populations. Studies have confirmed that microdissection increases the specificity of signals obtained in downstream protein analysis [206-209]. Laser-capture microdissection is particularly vital in the molecular profiling of normal and malignant tissue because of its utility in obtaining pure cell populations.

##### 3.2.3.5. Proteins quantification

Because most disease associated markers are not exclusively expressed in either the disease or the “healthy” state, quantification of protein expression differences must be included in marker identification strategies. The quantification strategies used in combination with MS based proteomics are often based on the introduction of stable isotopes into the samples, which can be done either by metabolic, chemical, or proteolytic labeling (Table 5).

###### SILAC

The most widely used metabolic labeling strategy is stable isotope labeling by amino acids in cell culture (SILAC). SILAC is simple and powerful because the label is introduced prior to protein purification but can mainly be applied to cells in culture. Quantitative proteomics using chemical and proteolytic labels is, in contrast to SILAC, sensitive to variations in protein purifications between the compared samples because the labels are introduced after protein purification.

###### ICAT

Isotope-coded affinity tags (ICAT) use stable isotope labeling to perform quantitative analysis of paired protein samples. It consists of a reactive group, which reacts with cysteine residues, a linker containing the stable isotopes and a biotin tag for purification of labeled peptides [210]. Both samples are mixed, digested with trypsin, fractionated by avidin affinity chromatography and then these differentially tagged peptides are scanned in a mass spectrometer. Spectral peak analysis in single mass spectrometric (MS) mode of the isotopically resolved peptides from the two different sources enables quantitation of the relative amounts of the peptide and hence the protein levels. One weakness of ICAT is that only cysteine containing peptides can be labeled. Approximately 10% of proteins do not have cysteine, therefore they will not be detected by ICAT.

###### iTRAQ

In the iTRAQ system, the tags react with the N termini of the peptide and lysine residues, thus tagging all peptides [211]. iTRAQ contains a set of four isobaric reagents and therefore can analyze up to four protein samples at one time. After trypsin digestion, samples are labeled with four independent iTRAQ reagents and analyzed by MS. The intensity of each of these peaks represents the quantity of small reporter group fragments and thus represents the quantity of a peptide sample. Peaks in the spectrum graph are used to identify peptide sequences and therefore protein sequences. A comparative analysis of iTRAQ and ICAT suggests that the information generated by the two methods is complementary. ICAT is preferred for low abundant proteins including signaling molecules; however, overlapping peaks in the MS spectrum can compromise the quality of results. On the other hand, apart from nonspecific nature of labeling, iTRAQ requires lengthy sample processing separately that increases the chances of experimental variation [212].

###### ^18^O

Heavy oxygen (^18^O) can be introduced into peptides through proteolytic labeling by digesting the proteins in the presence of H_2_^18^O using trypsin, Lys-C, or Glu-C, which introduces one or two ^18^O molecules into the peptides [213].

###### MudPIT

MudPIT is an approach that uses multidimensional high-pressure liquid chromatography separation, tandem mass spectrometry and database searching [214]. MudPIT permits a rapid and simultaneous separation and identification of proteins and peptides in a complex mixture without the need for pre- or post-separation labeling, which is not possible in ICAT or iTRAQ [215]. The complex protein mixture is digested with a specific protease, peptide fragments are separated in parallel by two dimensional liquid chromatography (strong cation exchange column and reverse phase column). Eluted peptides are identified by tandem mass spectrometry. The technique is extremely sensitive and reproducible. One of the major weaknesses of MudPIT is in identifying quantitative differences in protein expression across protein mixtures [216].

##### 3.2.3.6. Immuno-enrichment

The most selective and sensitive methods for the enrichment of low abundant analytes in proteome analyses use highly selective capture molecules. Immunoprecipitation, co-immunoprecipitation or pull-down assays have been set up to enrich single proteins or protein complexes from highly complex samples followed by direct MS-based quantification or by proteolytic cleavage and identification of peptides via peptide mass fingerprint or MS/MS-based methods [217-220]. Other approaches use antibody phage display technology which is a strategy used to isolate tumor specific antibodies able to bind their cognate antigens in the cellular context for therapeutic uses [221-224]. For antibody phage display, antibody fragments are fused to the pIII minor capsid protein and displayed at the surface of filamentous phage M13. Repertoires of antibody variable (V) domains can be generated and used to construct large libraries of human scFv, Fab, or single domain antibody, which can then be used to generate panels of antibodies to virtually any antigen [225,226]. Direct selection of tumor specific antibodies from phage display human antibody libraries on tumor cells provides an approach for generating large panels of human antibodies that recognize tumor specific markers [225,227-231]. These tumor specific antibodies can be used to immunoprecipitate their tumor antigen for identification by MS, allowing a reduction of the sample complexity before MS based protein identification.

#### 3.2.4. Protein array

In a basic sense, protein arrays consist of immobilized protein in a defined area. Protein microarrays were first described by MacBeath and Schreiber in 2000, and the number of publications involving this technology is rapidly increasing [232]. Miniaturized microspot assays are becoming increasingly popular for protein-protein interaction analysis and protein profiling. Each array spot contains homogeneous or heterogeneous capture agents such as antibodies [233-235], aptamers, recombinant proteins or peptides [232,236], cell or phage lysates [237], or drugs immobilized at high spatial density on a solid surface to selectively extract target proteins from complex mixtures, including serum and cell lysate samples. They are the protein analog of cDNA arrays. However, they are technically more difficult to make because proteins are more complex in their composition, protein folding, denaturation, aggregation, and multimerization. Protein-detecting microarrays are typically used for two different types of analysis: (a) Determining the abundances of target proteins in a complex mixture through highly specific antigen-antibody interactions [238]; and (b) Providing information on the functions of target proteins through protein-protein interactions, receptor-ligand interactions, enzymatic activities, and other methods [239-245]. Protein arrays are being used for drug discovery, biomarker identification and molecular profiling of cellular material [236,246-248]. There are currently two classes of protein microarrays used in human sample research: Forward-phase protein microarrays (FPPAs) and reverse-phase protein microarrays (RPPAs) (Table 5).

##### FPPA

Forward-phase arrays use immobilized antibodies as bait to capture specific antigens within a heterogeneous mixture. As bait antibodies incubate with a test sample, antigens of interest become bound to their corresponding antibodies. The antigens of interest are then detected and visualized by a second “sandwich” antibody. The disadvantage of FPPAs is the requirement for 2 antibodies for the identification of any particular antigen. Therefore, the antigens of interest must be in conformational states allowing the binding of two distinct antibodies.

The most commonly used microchips are planar antibody microarrays, where well-characterized antibodies are immobilized to capture the proteins of interest. The wider application of protein arrays in biomedical research is still limited, partly because of the cost of producing and immobilizing antibodies and the limited availability of antibodies with high specificity and high affinity for their target. Recently, new strategies have been developed to solve these problems such as the use of very stable and available single domain antibodies (sdAb) [249]. Additionally, the difficulties associated with preserving proteins in their biologically active conformation before analysis with protein arrays further limits the application of this technology as a routine proteomic strategy. Nevertheless, protein-array platforms became an attractive profiling approach among many proteomics technologies [245,250-253] because of the promise of large scale analysis that can be performed with relatively low amount of sample, technical ease and high throughput [254,255]. Microbead-based protein arrays are based on the interaction between surface (polystyrene microspheres) attached capture molecules and proteins of cell lysates. The surface bound complexes on the microbeads are interrogated by flow cytometry. This technique is suitable to detect protein-protein, nucleic acid–protein, and nucleic acid-nucleic acid interactions. Multiplexing is achieved by either using different sized microbeads or color coding that is readable by a laser-induced fluorescent detection system [256]. This latter approach is suitable for the analysis of up to a hundred different bead-bound antibodies and/or proteins in a complex mixture.

##### RPPA

Reverse-phase protein microarrays have been introduced by Paweletz *et al.* in 1998 [257]. The name “reverse-phase” is used because cell lysates are immobilized in the solid phase and are probed with an antibody. An array can be composed of many patient samples in a dilution curve format that allow quantitation [258]. Moreover, multiple lysates representing normal invasive cell populations may be printed in parallel on the same array. There are many advantages of reverse-phase protein arrays to analyze cancer-related protein networks. First, RPPAs have higher throughput capabilities. Reverse-phase protein microarrays require low sample volume (approximately 2 nL per spot), enabling researchers to print hundreds of patient samples onto a single array slide. In addition, the low volume requirement allows for analysis of often-limited patient biopsy material. The high-throughput nature of RPPAs is also necessary for the real-time analysis of patient tissue.

##### NAPPA

The next advancement in protein microarrays was development of high-density, self-assembling protein microarrays, based on the concept of the nucleic-acid programmable protein array (NAPPA) [251,252,254]. The concept is to synthesize proteins on the high-density chip using spotted cDNA and a T7-coupled rabbit reticulocyte lysate *in vitro* transcription-translation (IVTT) system [251]. Translated proteins contain a C-terminal glutathione S-transferase (GST) tag, which is used to capture co-printed anti-GST antibody. NAPPA represents a crucial step in addressing many of the concerns related to manufacturing limitations (e.g., density of printing, reproducibility, and quality of immobilized proteins).

To resume, microarrays are useful for high throughput analysis of candidate biomarkers in patient samples. However, the method has a limited role in discovery based identification of novel biomarkers where other platforms, such as MS, are better suited. Despite the technological advances, protein microarrays still suffer from skepticism and criticism. At present, protein arrays remain an emerging technology [259,260] that requires further technological developments and refinements but have great potential as complementary approaches to other profiling platforms.

#### 3.2.5. Surface plasmon resonance

Surface plasmon resonance (SPR) is one of the most sophisticated methods used today to detect and quantify biomolecular interactions in real time in a nondestructive manner without any labeling requirement [261] (Table 5). Capture agents are immobilized on a gold surface, and the change in the reflection angle of light is used to quantify the number of unlabeled target molecules captured on the surface [262]. In conventional SPR systems, a single channel is available within a single experiment. Thus, miniaturization and parallelization of SPR apparatus have been elaborated to perform multiple measurements in a single experiment. Recently, a SPR imaging technique was developed to improve throughput in SPR-based detection of molecular interactions. For instance, the S-protein-S-peptide interaction was examined using an array composed of five different peptides, including S-peptide, by determining an association rate, a dissociation rate, and an equilibrium association constant [263]. SPR imaging methods can now monitor hundreds of biomolecular interactions in real time simultaneously, and are suitable for unqualitative screening and quantitative kinetics experiments [264]. The integration of SPR and MS has proven useful in the analysis of biomolecular interaction patterns, including drug candidates, enzyme inhibitors, DNA binding proteins, disease markers, peptide sequences, and post-translational modification [265-268].

#### 3.2.6. Tissue microarray

Tissue microarray (TMA) technology was first described by Wan *et al.* in 1987 [269]. However, it was not until 10 years later, when Kononen *et al.* developed a device that could rapidly and reproducibly produce quality TMAs [240] that this technique emerged. The key benefit underlying TMA technology is the ability to assay hundreds of patient tissues arrayed on a single microscope slide. In its most common form, a core of tissue is lifted from a formalin-fixed, paraffin embedded sample and placed in a predrilled hole in a paraffin recipient block. On sectioning, each sample is represented as a small (0.6- to 2-mm diameter) histologic section arrayed in a grid that allows easy linkage to clinicopathologic data. The result is a single slide that contains samples from 40 to 800 patients (depending on core size). Other researchers have adapted TMA technology to frozen tissues [270], cell lines [271,272], and needle biopsies [273]. TMA provide several benefits. Each TMA uses only a small core from the donor blocks, each block can be used in dozens (or potentially hundreds) of newly created TMAs. Second, TMAs can drive significant cost savings both in terms of reagents and technician time required to stain one slide instead of hundreds. Third, because of the inherent efficiency in processing hundreds to thousands of tumors at one time, TMAs can dramatically increase the number of tumors that can be analyzed compared with traditional whole-section studies. TMAs are arrayed on a single slide, all of the tumor specimens are stained consistently, at the same time, under the same conditions, and with exactly the same antibody dilution. However, because TMAs examine only a fraction of the tumor that is analyzed using traditional methods, many researchers were initially concerned that TMA cores would not adequately assess biomarkers that exhibited tissue heterogeneity. Subsequently, multiple groups have demonstrated strong correlations between TMA histospots and whole-tissue sections [274,275]. Although the size of TMA histospots presented challenges to assessing tumor heterogeneity, they also provided a new opportunity for developing automated methods of analysis. Indeed, histospots are sufficiently small to allow a rigorous molecular quantification. Because TMAs are prevalidated by a pathologist during construction, automated systems would only have to assess staining intensity. Automated analysis permits the quantification of biomarkers in a way that matches their biologic expression. The last 10 years have provided an opportunity to invent and refine new techniques in production, staining, and analysis that will help TMA technology with the big challenge of discovery of biomarkers. TMAs are ideally suited to rapidly triage hundreds or thousands of potential biomarkers, permitting researchers to focus on a few likely candidates [276]. It has become an attractive validation strategy and is also sometimes described as a proteomics technique. This type of validation of potential novel biomarkers, including PM proteins, relies on access to large numbers of biological samples, e.g., biopsies of primary tumors and metastases collected and stored for research at hospitals.

### 3.3. Glycosylated Proteins

Glycosylation is the most common, being present in ∼50% of the total number of proteins [65]. Cancer cells frequently display glycoproteins with increased branching of the glycan structures and/or altered expression levels compared with normal cells [82]. An increase in the branching creates additional sites for terminal sialic acid residues, negatively charged acidic sugars that can be recognized by lectins [277]. The glycan structure or expression level of many PM glycoproteins may be altered, alterations may also occur on secreted glycoproteins and serve as biomarkers for early detection of cancers [82,278-280]. Tumor markers in current clinical use, such as carcinoembryonic antigen, prostate-specific antigen, HER-2, and mucins (e.g., CA 19.9, CA 125, and CA 15.3), are all glycoproteins that are either membrane-associated or secreted to the serum [280-282].

Glycoproteomics usually includes enzymatic digestion of the glycoprotein-containing samples to generate peptides and glycopeptides. The glycopeptides are then enriched using selective chromatographic methods, typically using immobilized lectins, hydrophilic interaction LC, titanium dioxide, or graphite [283-291]. Lectins, e.g., concanavalin A, differ in their specificity and selectivity toward glycan compositions. The glycopeptides recovered by one or a combination of enrichment methods are then analyzed using MS, which can be used to obtain spectra of intact glycoproteins, glycopeptides, or released glycans [140,292]. The challenge with quantitative and qualitative analysis of glycopeptides is that it is not always possible to obtain glycopeptides with just one glycosylation site, and not all glycosylation sites are necessarily occupied by glycan moieties, whereas others may be partially occupied. Exploiting differences in glycosylation between malignant and healthy tissues likely affords excellent opportunities to identify sensitive and specific cancer biomarkers [92,284,293].

## 4. Conclusions

Application of genomic and proteomic technologies have led to the identification of many hundreds to thousands of biomarker candidates for several diseases. The identification and characterization of tumor specific markers remains a major goal in both understanding the cellular transformation observed in cancer and in developing targets for the molecular therapy of cancer. Molecules that are tumor-specific or overexpressed in cancer are likely to have functional roles that participate in cellular transformation and migration. Targeting of such molecules can result in an anti-tumor effect and therefore might be of interest for cancer therapy. Of particular interest within the spectrum of tumor-specific and overexpressed molecules are those located at the cell surface, since they are readily accessible and can be used to target cancer cells with highly specific ligands like mAbs. A difficulty of protein expression profiling arises from the unpredictable rate of protein degradation. Proteins act mostly as effector molecules with a short life time and their degradation is influenced by many parameters such as size, structure, composition, co- and post-translational modifications, *etc.* Changes can also be caused by an altered reaction environment such as pH, salt concentration, hydrophobicity, or by analysis-related artifacts that are generated during sample processing.

New challenges arise in large scale proteomic profiling when dealing with complex biological mixtures such as mammalian cell lysate. Identification of large numbers of proteins from complex biological samples is a continuing challenge in the area of quantitative proteomics. However, the sample complexity can be effectively reduced with corresponding increases in protein identification using various methods. In the near future, the refinement and possible combination of these emerging techniques will likely lead to the identification of a large panel of new biomarkers and tumor antigens. Hopefully these new markers will then be used to develop efficient diagnostic procedures and relevant immunotherapeutic approaches against a large variety of cancers.

## Figures and Tables

**Table 1.** Monoclonal antibodies approved by Food and Drug Administration (US) or European Medical Agency (EU).

**Product**	**Type**	**Target**	**Indications**	**Date of approved**
Rituximab (Rituxan)	Chimeric	CD20	Non-Hodgkin's lymphoma	1997 (US)
				1998 (EU)
Trastuzumab (Herceptin)	Humanized	HER2	Metastatic breast cancer	1998 (US)
				2000 (EU)
Gentuzumab (Mylotarg)	Humanized, (coupled to calicheamicin)	CD33	Acute myeloid leukemia	2000 (US)
Alemtuzumab (Campath)	Humanized	CD52	Chronic lymphocytic leukemia	2001 (US)
				2001 (EU)
Ibritumomab tiuxetan (Zevalin)	Chimeric (^90^Y radiolabelled)	CD20	Non-Hodgkin's lymphoma	2002 (US)
				2004 (EU)
Tositumomab (Bexxar)	Murine (^131^I radiolabelled)	CD20	Non-Hodgkin's lymphoma	2003 (US)
Cetuximab (Erbitux)	Chimeric	EGFR	Metastatic colorectal cancer	2004 (EU)
Bevacizumab (Avastin)	Humanized	VEGF-A	Metastatic colorectal cancer	2004 (US)
				2005 (EU)
Panitumumab (Vectibix)	Human	EGFR	Metastatic colorectal cancer	2006 (US)
				2007 (EU)
Catumaxomab (Removab)	Hybrid rat and mouse (trifunctional bispecific)	EpCam	Malignant ascites	2009 (EU)
Ofatumumab (Arzerra)	Human	CD20	Chronic lymphocytic leukemia	2009 (US)

**Table 2.** Main tumor antigens used in clinical studies using mAb therapy.

**Target**	**Antigen class/description**	Number of mAbs *
EpCam (Epithelial cell adhesion molecule)	Cellular adhesion	17
EGFR (Epidermal growth factor receptor)	Growth factor receptor	12
CD20	CA2+ channel	10
MUC1 (Mucin 1)	Mucin	10
HER2 (Human epidermal growth factor receptor 2)	Growth factor co-receptor	9
CEA (Carcinoembryonic antigen)	Oncofetal glycoprotein, cellular adhesion	9
CD22 (Siglec-2)	Sialoadhesin, sialic acid-binding immunoglobulin-like lectine	6
CD33 (Siglec-3)	Sialoadhesin, sialic acid-binding immunoglobulin-like lectine	6
LEWIS Y	Carbohydrate	6
PSMA (Prostate-specific membrane antigen)	Glycoprotein with folate hydrolase and NAALADase activities	6
TAG-72 (Tumor-associated glycoprotein 72)	Mucin like glycoprotein	5
CD30 (TNFRSF1)	TNF receptor super family	4
CD19	B-lymphocyte antigen	3
CD44V6	Adhesion molecule	3
CD56 (NCAM)	Cellular adhesion	3
GD2 ganglisoside	Glycosphingolipid	3
GD3 ganglisoside	Glycosphingolipid	3
HLA-DR10 (Human leukocyte antigen-DR)	MHC class II receptor	3
IGF1R (Insulin-like growth factor 1 receptor)	Tyrosine kinase receptor	3
TAL6 (Tumor-associated antigen L6)	Members of the transmembrane-4 superfamily	3
TRAILR2 (Tumor-necrosis factor-related apoptosis-inducing ligand receptor)	Member of the tumor necrosis factor family	3
VEGFR2 (Vascular endothelial growth factor receptor 2)	Angiogenic growth factor receptor	3
CD152 (CTLA4)	Negative regulator of T cell activation	2
Unknown		11

* mAbs in clinical studies between 1980 and 2005 [28].

**Table 3.** Overview of different vaccination strategies.

**Vaccine Type Name**		**Phase**	**Tumor**	**Antigen**	**References**
Viral vectors	PSA-TRICOM	II	Prostate	PSA	[31-34]
	PANVAC-VF	III	Pancreatic	CEA, MUC1	[35-37]
	TG4010	II	Breast, prostate, lung	MUC1, IL2	[38-40]
Peptides	Provenge	III	Prostate	PAP	[1,2]
	Oncophage	III	Melanoma, Renal	HSPg96	[42,43]
	Stimuvax	II	Lung	Extracellular core peptide of MUC1	[41]
Tumor cells or tumor-cell lysates	OncoVAX	III	Colon	Irradiated tumor cells	[44]
	Renial	III	Renal	Lysate of autologous tumor cells	[44,45]
	GVAX	III	Prostate	Irradiated humn prostate cancer cell lines LNCaP and PC-3	[35]
RNA	mRNA from Pca cell lines	II	Prostate	PSA	[57]

**Table 4.** Main cancer biomarkers and their applications.

**Biomarker**	**Cancer type**	**Clinical use in diagnosis**	**Clinical use in therapy**
CEA	Colon	Monitoring	Passive and active therapy
Alpha-fetoprotein	Germ-cell hepatoma	Staging	*not used*
CA125	Ovarian	Monitoring	Passive therapy
EGFR	Colon	Prognosis	Passive therapy
KIT	Gastrointestinal	Diagnosis	Molecular therapy (Imatinib)
Thyroglobulin	Thyroid	Monitoring	*not used*
PSA	Prostate	Screening and monitoring	Passive and active therapy
CA15-3	Breast	Monitoring	*not used*
CA27-29	Breast	Monitoring	*not used*
Cytokeratins	Breast	Prognosis	*not used*
Oestrogen and progesterone receptor	Breast	Prognosis	Hormonotherapy
HER2	Breast	Monitoring	Passive and active therapy
Fibrin/FDP	Bladder	Monitoring	*not used*
Mucin 1	Glandular epithelial origin	Diagnosis, monitoring	Passive and active therapy
CA19-3	Pancreatic	Monitoring	*not used*

**Table 5.** Methods for protein expression studies.

**Methods**	**Number of proteins**	**Advantages**	**Disadvantages**
ELISA	One	Well established, sensitive, specific, wide applicability	Separate assay for each protein
Western blotting	One	Wide applicability	Poor reproducibility
2D-Gel electrophoresis	Few thousand	Small costs, possibility of screening	Time-consuming
IHC	One	Determination of protein localization	Separate assay for each protein
TMA	One	Analysis of multiple samples	Problem of small tissue spot
Planar antibody array	Few hundred	Small sample volume, multiplexing capabilities, sensitive	Poor reproducibility, cross reactivity, labeling
RPPA	Few thousand	Small sample volume, multiplexing capabilities	Cross reactivity
Bead array	10–20 (max 100)	Multiplexing, small sample volume, wide applicability	Cross reactivity
MALDI-SM	Few thousand	Small sample volume, wide applicability, screening	Poor reproducibility, time-consuming
SELDI-SM	Few thousand	Small sample volume, screening	Low sensitivity, time-consuming
SPR	One	High sensitivity, small sample volume, no labeling	No screening, time-consuming
ICAT	Few thousand	Protein quantification of low abundant proteins	Only cysteine containing peptides can be analyzed (90%)
iTRAQ	Few thousand	Protein quantification	Lengthy sample processing separately
MudPIT	Few thousand	Protein identification and quantification, no labeling	No quantitative analysis
